# Exercise Mode in Heart Failure: A Systematic Review and Meta-Analysis

**DOI:** 10.1186/s40798-022-00549-1

**Published:** 2023-01-09

**Authors:** Jamie Edwards, Nesan Shanmugam, Robin Ray, Fadi Jouhra, Jennifer Mancio, Jonathan Wiles, Anna Marciniak, Rajan Sharma, Jamie O’Driscoll

**Affiliations:** 1 School of Psychology and Life Sciences, Canterbury Christ Church University, North Holmes Road, Canterbury, Kent, CT1 1 QU UK; 2 Department of Cardiology, St George’s Healthcare NHS Trust, Blackshaw Road, Tooting, London, SW17 0QT UK

**Keywords:** Heart failure, HFpEF, HFrEF, Exercise training, Exercise mode

## Abstract

**Background:**

Optimising exercise prescription in heart failure (HF) with a preserved (HFpEF) or reduced (HFrEF) ejection fraction is clinically important. As such, the aim of this meta-analysis was to compare traditional moderate intensity training (MIT) against combined aerobic and resistance training (CT) and high-intensity interval training (HIIT) for improving aerobic capacity (VO_2_), as well as other clinically relevant parameters.

**Methods:**

A comprehensive systematic search was performed to identify randomised controlled trials published between 1990 and May 2021. Research trials reporting the effects of MIT against CT or HIIT on peak VO_2_ in HFpEF or HFrEF were considered. Left-ventricular ejection fraction (LVEF) and various markers of diastolic function were also analysed.

**Results:**

Seventeen studies were included in the final analysis, 4 of which compared MIT against CT and 13 compared MIT against HIIT. There were no significant differences between MIT and CT for peak VO_2_ (weighted mean difference [WMD]: 0.521 ml min^−1^ kg^−1^, [95% CI] =  − 0.7 to 1.8, *P*_fixed_ = 0.412) or LVEF (WMD: − 1.129%, [95% CI] =  − 3.8 to 1.5, *P*_fixed_ = 0.408). However, HIIT was significantly more effective than MIT at improving peak VO_2_ (WMD: 1.62 ml min^−1^ kg^−1^, [95% CI] = 0.6–2.6, *P*_random_ = 0.002) and LVEF (WMD: 3.24%, [95% CI] = 1.7–4.8, *P*_random_ < 0.001) in HF patients. When dichotomized by HF phenotype, HIIT remained significantly more effective than MIT in all analyses except for peak VO_2_ in HFpEF.

**Conclusions:**

HIIT is significantly more effective than MIT for improving peak VO_2_ and LVEF in HF patients. With the exception of peak VO_2_ in HFpEF, these findings remain consistent in both phenotypes. Separately, there is no difference in peak VO_2_ and LVEF change following MIT or CT, suggesting that the addition of resistance exercise does not inhibit aerobic adaptations in HF.

**Graphical Abstract:**

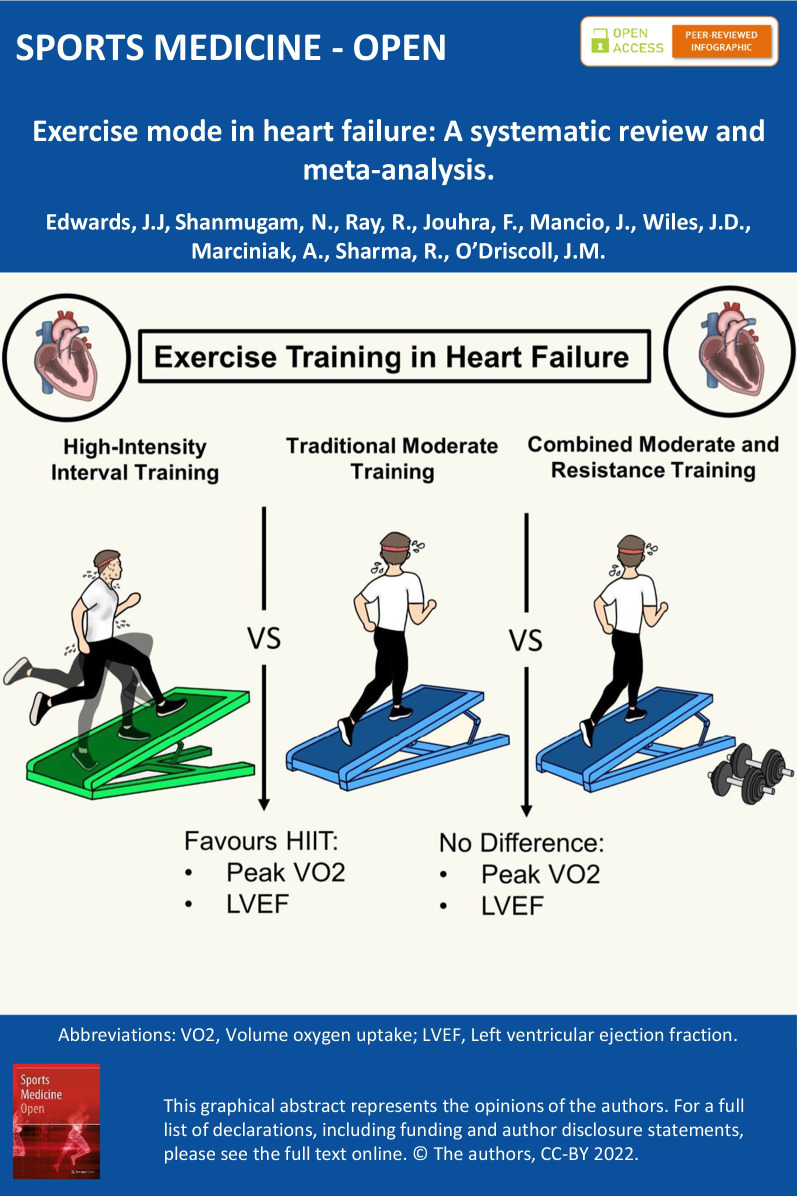

**Supplementary Information:**

The online version contains supplementary material available at 10.1186/s40798-022-00549-1.

## Key Points

- High-intensity interval training (HIIT) is more effective than moderate intensity training (MIT) for improving cardiorespiratory fitness and cardiac function in heart failure patients.
- There is no difference in cardiorespiratory fitness and cardiac function between MIT alone, and in combination with dynamic resistance training (CT). This suggests that dynamic resistance exercise does not negatively affect cardiorespiratory adaptations.

## Introduction

Exercise training (ET) is well established as an effective interventional strategy in heart failure (HF), constituting a major component of cardiac rehabilitation practices across clinics globally [1, 2]. Specifically, ET has been consistently demonstrated to produce significant improvements in various clinically important parameters, including aerobic capacity (VO_2_) [3], quality of life [3], and even cardiac function [4] and structure [5]. These adaptations have been demonstrated to translate into improvements in clinical outcomes, with significant reductions in all-cause and cardiovascular hospitalisation and, although the data remain more uncertain, all-cause mortality [6, 7]. Thus, given the pivotal role of ET in HF, establishing optimal exercise prescription practices with consideration of HF phenotype is essential.

Current exercise-based cardiac rehabilitation programmes typically employ low to moderate intensity aerobic training (MIT) regimes. Indeed, a plethora of research trials have demonstrated the effectiveness of MIT in HF; however, outpatient adoption and adherence rates are low, especially amongst women [6, 8, 9]. Moreover, very little research has investigated the comparative efficacy of MIT alone against MIT combined with resistance training (CT), which remains an important comparison to further optimise exercise prescription in HF. As discussed in the current guidelines [10], the addition of resistance training is important for the maintenance of muscle mass in an ageing HF population vulnerable to sarcopenia; however, whether this addition impacts adaptations in VO_2_ is not entirely clear.

There is also growing clinical interest in the application of high-intensity interval training (HIIT), a time-efficient and well-received exercise mode in cardiac rehabilitation settings, which appears to produce similar, or greater benefits to that observed following MIT [11, 12]. However, the current comparative literature between MIT and HIIT in HF overlooks the fundamental clinical differences between the two broad HF phenotypes, heart failure with a reduced (HFrEF) or preserved (HFpEF) ejection fraction. As such, there is poor understanding of the different effects of MIT and HIIT across the same parameters for both HFrEF and HFpEF [11].

As such, we aimed to perform a systematic review and meta-analysis of randomised controlled trails to establish the optimal exercise prescription in HF, comparing the effects of MIT against CT and HIIT on peak VO_2_ and various other clinically relevant parameters. In addition, we aimed to perform the first sub-group analysis on the differences in efficacy between MIT and HIIT in HFrEF and HFpEF separately.

## Methods

This systematic review and meta-analysis was performed in accordance with the PRISMA guidelines [13]. PubMed (Medline), the Cochrane library and Web of Science were systematically searched for research trials reporting the effects of MIT against CT or HIIT on peak VO_2_ in HFpEF or HFrEF. Trials published between 1990 and May 2021 were considered. The search strategy included combinations of the relevant medical subject heading (MeSH) terms, text words and word variants for exercise, physical activity, cardiac rehabilitation, heart failure, HFpEF, HFrEF, diastolic heart failure, preserved ejection fraction and reduced ejection fraction, with the Boolean search terms ‘OR’ and ‘AND’ (see Additional file 1). Reference lists of relevant articles and reviews were hand searched for additional reports and where relevant, corresponding authors were contacted to ascertain whether non-published data were available or in the pre-print stage.

### Study Eligibility, Outcome Measures and Data Collection

Randomised controlled trials of adults (≥ 18 years) reporting peak VO_2_ following two separate exercise interventions with an eligibility criterion of 4 weeks to 6 months in duration were considered. Secondary outcomes of interest were: left ventricular ejection fraction (LVEF), end-diastolic volume (EDV), the ratio of early to late diastolic peak blood flow velocity (E/a ratio) and the ratio between early mitral inflow velocity and early mitral annular velocity (E/e′). Studies that did not report data for both separate exercise interventions were excluded. MIT and HIIT are defined according to the EXPERT tool [14]. One seemingly suitable study was excluded due to not meeting the intensity thresholds to be considered HIIT [15], one did not categorically fit the criteria for MIT [16], and another included concurrent exercise modes with the potential for confounding effects [17]. To be classified as HIIT, the intensity metrics were required to fall within the categories of ‘High-intensity, vigorous effort’ or ‘Very hard effort’ in the EXPERT tool [14]. CT was determined as MIT performed concurrent to any resistance training intervention. HFpEF and HFrEF are defined according to the respective studies individually, and HF severity according to the New York Heart Association (NYHA) classifications. We defined an immediate adverse event as an acute event that occurred during, or immediately after, an exercise session, excluding any later follow-up event data.

Two authors (JE and JOD) separately screened all papers for eligibility. Any inconsistency or confliction was discussed by the researchers and a consensus was reached. All studies were initially screened by title and abstract, and subsequently by full text if they met the relevant inclusion criteria. Following study recruitment, the relevant data of all included studies were extracted independently by the two researchers for synthesis. If more than one study was published for the same cohort, the study containing the most comprehensive and relevant information was included to avoid overlapping populations. Authors were contacted when the methodology indicated the collection of data, but such data were not reported.

### Study Quality Assessment

Study quality and risk of bias was evaluated using the TESTEX scale [18]. TESTEX is a validated 15-point (12 item) tool designed for the specific application to exercise training studies. Two researchers (JE and JOD) independently scored all eligible articles and any disputes in the quality analyses were resolved via consensus. Detailed TESTEX scoring for each study can be found in the Additional file 1 (Table S1 and Table S2).

### Statistical Analysis

All statistical analysis was performed using the statistical software Comprehensive Meta-Analysis (Comprehensive Meta-Analysis version 3, Biostat, Englewood, NJ, USA). As all outcomes were measured on the same scale across all studies, weighted mean difference (WMD) with 95% confidence intervals (CI) was calculated. Effect sizes were calculated based on the WMD (change score) between baseline and follow-up measures to establish the comparative efficacy between MIT and CT, and MIT and HIIT [19]. Analyses of effect sizes were conducted for HF as a collective and subsequently dichotomised into HFpEF and HFrEF subgroups if data allowed. Multiple meta-regression analyses were also conducted to ascertain if any effect moderator variables influenced the primary outcomes. The moderators assessed independently were: age, body mass index (BMI) and intervention duration (weeks). Statistical inter-study heterogeneity was tested alongside the pooled analysis and reported as the I^2^ statistic. A significance threshold of > 40% was applied to the *I*^2^ statistic [20]. Once past this threshold, random-effects analysis was applied and a post hoc Egger’s test was performed to assess the presence of publication bias [21]. Sensitivity analysis was performed for the primary outcomes using the in-built CMA ‘one-study removed’ analysis method, which did not significantly influence any of the overall effect sizes. Statistical significance of the pooled analysis was determined with a *P* value of < 0.05 and a *Z*-value of > 2.

## Results

**Fig. 1:**
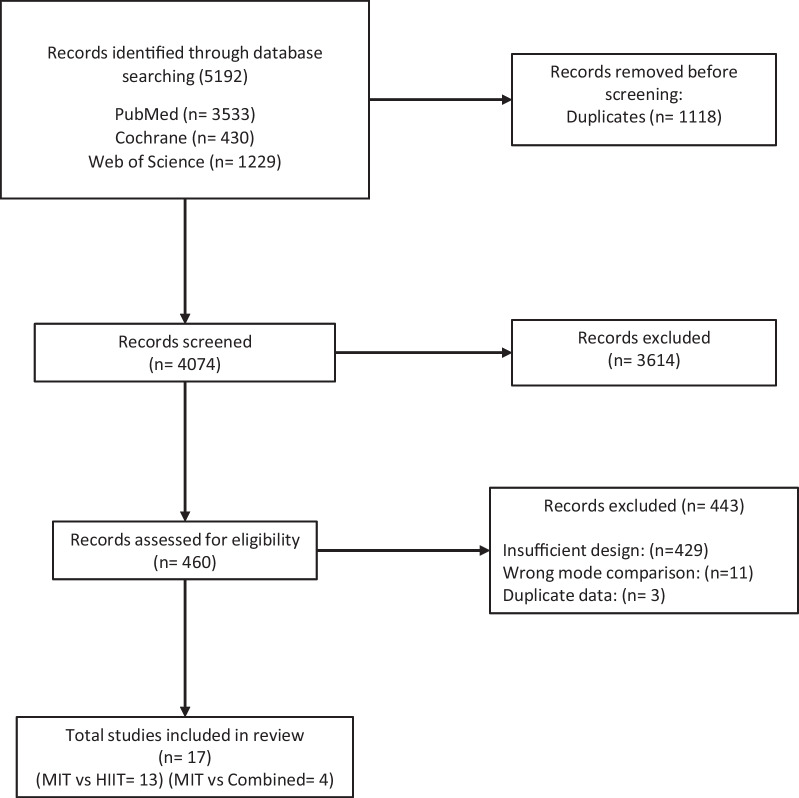
PRISMA systematic review and meta-analysis flowchart. MIT, moderate intensity training; HIIT, high-intensity interval training

Figure 1 represents the PRISMA systematic review flowchart. Following all exclusions, 17 studies were analysed, 4 of which compared MIT against CT (150 participants) and 13 compared MIT against HIIT (565 participants). Detailed TESTEX scoring of each study can be found in the Additional file 1 (Tables S1 and S2). Study training and participant characteristics for MIT versus CT and MIT versus HIIT can be found in Additional file 1: Tables S1, S2, S3 and S4, respectively.

While we found that some large studies (Ellingson et al. [22] and Mueller et al. [23]) reported higher adverse events during the 12-month follow-up in HITT patients versus MIT patients, we found minimal immediate adverse events and zero deaths/serious events during or following an ET session. The acute safety data of MIT, CT and HIIT in both HFrEF and HFpEF are presented in Tables 1 and 2.

**Table 1:** MIT versus CT training characteristics

Study	Groups	Design	Country	Exercise training mode	Exercise Intensity	Exercise frequency (days p/w)	Intervention duration (weeks)	Adverse events
Laoutaris et al. [24]	MIT	RCT	Greece	Aerobic training (cycling)	70–80% MHR	3	12	0
	Combined	RCT	Greece	Combined aerobic (cycling) and dynamic RT	Aerobic (30 min) = 70–80% MHR, RT (15 min) = 50% 1RM, IMT (20 min) = 60% SPImax	3	12	0
Beckers et al. [25]	MIT	RCT	Belgium	Aerobic (treadmill, bicycle, stair, arm-cycling, half recumbent or recline cycling)	90% HR achieved at VT2 (60 min)	3	6 months	0
	Combined	RCT	Belgium	Aerobic (treadmill, bicycle, stair, arm-cycling, half recumbent or recline cycling) and dynamic RT	50–60% 1RM (23–40 min) and 90% HR achieved at VT2 (10–37 min)	3	6 months	0
Mandic et al. [26]	MIT	RCT	New Zealand	Aerobic training (treadmill and cycling)	50–70% HRR (30 min)	3	12	0
	Combined	RCT	New Zealand	Aerobic training (treadmill and cycling) and dynamic RT (chest press, bicep curl, etc.) on weight machines	50–70% HRR (30 min), 50–70% 1RM	3	12	1 (AF episode)
Servantes et al. [27]	MIT	RCT	Brazil	Aerobic (walking)	HR corresponding to anaerobic threshold	3–4	3 months	0
	Combined	RCT	Brazil	MIT (walking) and strength training (upper and lower limb)	30–40% 1RM	3–4	3 months	0

MIT, Moderate intensity training; RCT, randomised controlled trial; RT, resistance training; MHR, maximal heart rate; 1RM, 1-repetition maximum; HRR, heart rate reserve; AF, atrial fibrillation

**Table 2:** MIT versus HIIT training characteristics

Study	Groups	Design	Country	Exercise training mode	Exercise intensity	Exercise frequency (days p/w)	Intervention duration (weeks)	Adverse events
Iellamo et al. [28]	MIT	RCT	Italy	Aerobic (uphill treadmill walking)	45–60% HRR (30–45 min)	2–5	12	0
	HIIT	RCT	Italy	Aerobic (uphill treadmill walking)	75–80% HRR (4 × 4 min intervals, by 2–4 times)	2–5	12	0
Ellingsen et al. [22]	MIT	RCT	Norway (9 centres)	MIT (treadmill or cycling)	60–70% MHR (47 min)	3	12	NR
	HIIT	RCT	Norway (9 centres)	HIIT (treadmill or cycling)	90–95% MHR (4 × 4 min) 38 min session	3	12	NR
Iellamo et al. [29]	MIT	RCT	Italy	Aerobic (uphill treadmill walking)	45–60% HRR (30–45 min)	3	12	0
	HIIT	RCT	Italy	Aerobic (uphill treadmill walking)	75–80% HRR (4 × 4 min intervals, by 2–4 times)	3	12	0
Besnier et al. [30]	MIT	RCT	France	MIT (cycling)	60% peak power output (30 min)	5	3.5	0
	HIIT	RCT	France	HIIT (cycling)	100% peak power output (Two 8-min blocks of 30 s max output and 30 s active rest)	5	3.5	0
Koufaki et al. [31]	MIT	RCT	UK	MIT (cycling)	40–60% peak VO_2_ (21–40 min)	3	24	1 (anxiety attack)
	HIIT	RCT	UK	HIIT (cycling)	100% peak power output (2 × 15 min bouts of 30 s max output with 1 min active rest between)	3	24	1 (syncope)
Wisløff et al. [32]	MIT	RCT	Norway	moderate continuous—uphill walking	70–75% peak HR	3	12	0
	HIIT	RCT	Norway	Aerobic interval training—uphill walking (4 × 4-min intervals)	90–95% peak HR	3	12	0
Dimopoulos et al. [33]	MIT	RCT	Greece	MIT (cycling)	50% WR peak (40 min)	3	12	NR
	HIIT	RCT	Greece	HIIT (cycling)	100% WR peak (30 s intervals and 30s rest for 40 min)	3	12	NR
Freyssin et al. [34]	MIT	RCT	France	MIT (cycling and treadmill)	HR corresponding VT1 (45 min)	5	8	0
	HIIT	RCT	France	AIT (cycling)	50% steep ramp test (30 s intervals for 40 min)	5	8	0
Fu et al. [35]	MIT	RCT	Taiwan	MIT (cycling)	60% HRR/VO_2_ peak	3	12	NR
	HIIT	RCT	Taiwan	HIIT (cycling)	80% HRR/VO_2_ peak	3	12	NR
Ulbrich et al. [36]	MIT	RCT	Brazil	MIT (uphill walking)	75% peak HR	3	12	0
	HIIT	RCT	Brazil	HIIT (uphill walking)	95% peak HR (3 min intervals with 3 min active recovery, 4–6 times)	3	12	0
Donelli da Silveira et al. [37]	MIT	RCT	Brazil	MIT (treadmill)	50–60% peak VO_2_	3	12	0
	HIIT	RCT	Brazil	HIIT (treadmill)	80–90% peak VO_2_	3	12	0
Angadi et al. [38]	MIT	RCT	USA	MIT (treadmill)	60–70% peak HR	3	4	0
	HIIT	RCT	USA	HIIT (treadmill)	80–85% peak HR	3	4	0
Mueller et al. [23]	MIT	RCT	Germany	HIIT (cycling)	80–90% HRR	5	12	NR
	HIIT	RCT	Germany	Aerobic (cycling)	35–50% HRR	3	12	NR

HIIT, High-intensity interval training; MIT, moderate intensity training; RCT, randomised controlled trial; MHR, maximal heart rate; HRR, heart rate reserve; WR, work rate; VO_2_, volume oxygen

### MIT versus CT

**Fig. 2:**
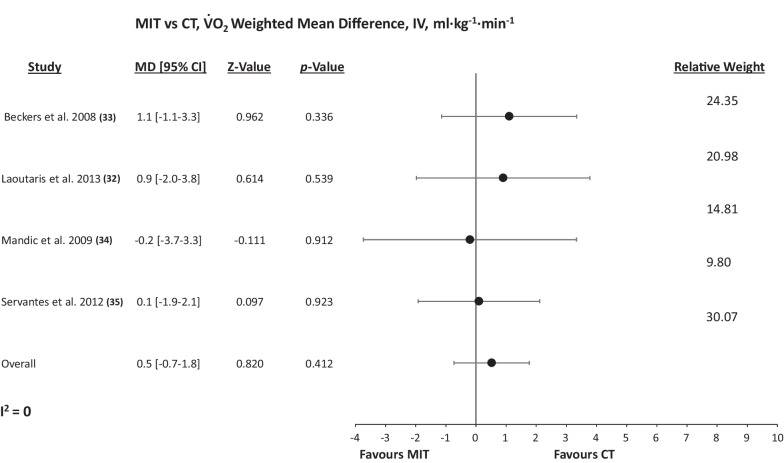
Random-effects meta-analysis of the weighted mean difference in peak VO_2_ between MIT and CT. MIT, Moderate intensity training; CT, combined training; MD, mean difference; VO2, peak oxygen uptake; IV, weighted mean difference

Figure 2 details the WMD in peak VO_2_ change following MIT and CT. There was no significant difference between the two modalities for peak VO_2_ change (WMD: 0.521 ml min^−1^ kg^−1^, [95% CI] =  − 0.7 to 1.8, *P*_fixed_ = 0.412). There was no significant heterogeneity (*P* = 0.885, *I*^2^ = 0%) or evidence of publication bias (*P* = 0.869, Additional file 1: Fig. S2). Due to insufficient HFpEF data, dichotomization by phenotype was not statistically possible. Furthermore, there was no significant difference between MIT and CT for LVEF (WMD: − 1.129%, [95% CI] =  − 3.8 to 1.5, *P*_fixed_ = 0.408). There were insufficient data for any further secondary outcome analysis.

### MIT versus HIIT

**Fig. 3:**
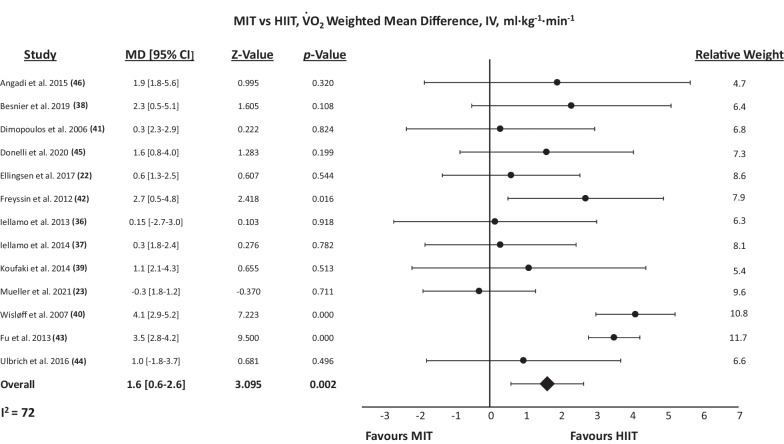
Random-effects meta-analysis of the weighted mean difference in peak VO_2_ between MIT and HIIT. MIT, Moderate intensity training; HIIT, high-intensity interval training; MD, Mean Difference; VO2, peak oxygen uptake; IV, weighted mean difference

Figure 3 presents the WMD in peak VO_2_ change following MIT and HIIT. HIIT produced improvements in peak VO_2_ to a significantly greater extent than did MIT (WMD: 1.62 ml min^−1^ kg^−1^, [95% CI] = 0.6–2.6, *P*_random_ = 0.002). There was statistically significant heterogeneity (*P* < 0.001, *I*^2^ = 72.1%), and the post hoc Egger’s test was statistically significant (*P* = 0.004), suggesting publication bias (Additional file 1: Fig. S1). When dichotomized by HF phenotype, HIIT was significantly more effective than MIT in HFrEF (WMD: 1.88 ml·min^−1^ kg^−1^, [95% CI] = 0.8–2.9, *P*_random_ = 0.001), but there was no significant difference observed in HFpEF (WMD: 0.44 ml min^−1^ kg^−1^, [95% CI] =  − 0.8 to 1.7, *P*_fixed_ = 0.485).

HIIT also produced increases in LVEF to a significantly greater extent than did MIT (WMD: 3.24%, [95% CI] = 1.7–4.8, *P*_random_ < 0.001). This statistical significance was consistent across both HFrEF (WMD: 3.88%, [95% CI] = 1.6–6.2, *P*_random_ < 0.001) and HFpEF (WMD: 2.3%, [95% CI] = 0.8–3.8, *P*_fixed_ = 0.002) separately. There was no significant difference between MIT and HIIT for EDV, *E*/*a* or *E*/*e*′.

### Moderator Analysis

The MIT versus CT meta-regression also demonstrated no significant effect of age (*B* =  − 0.0468, *P* = 0.7644 and *B* = 0.0818, *P* = 0.6530) or BMI (*B* =  − 0.3267, *P* =  − 0.5457 and *B* =  − 0.2262, *P* = 0.4840), with insufficient intervention duration data to perform such analysis.

The MIT versus HIIT meta-regression analysis demonstrated no significant effect of intervention duration (*B* =  − 0.018, *P* = 0.700), age (MIT group: *B* =  − 0.0705, *P* = 0.6229 and HIIT group: *B* = 0.1294, *P* = 0.3439) or BMI (BMI: *B* =  − 0.0308, *P* = 0.8200 and *B* =  − 0.0920, *P* = 0.5196).

## Discussion

For the purpose of optimising exercise prescription in HF, this meta-analysis compared the efficacy of MIT against CT and HIIT separately for improving peak VO_2_ as well as other various clinically relevant parameters. Importantly, our findings show that HIIT is significantly more effective than MIT for improving peak VO_2_ and LVEF in HF patients. When the analysis was dichotomized by HF phenotype, HIIT was significantly more effective than MIT for improving peak VO_2_ and LVEF in HFrEF, while LVEF, but not peak VO_2_, was significant in HFpEF. Separately, we found no significant differences between MIT and CT for peak VO_2_ or LVEF, suggesting that the application of resistance training in HF patients has no detrimental effects on aerobic adaptations following MIT.

Peak VO_2_ is a key prognostic marker in HF, with previous research reporting a 6% increase in peak VO_2_ to be associated with an 8% lower risk of HF hospitalisation and a 7% reduced risk of all-cause mortality [39]. While both MIT and HIIT appear effective, our findings indicate HIIT to be the superior mode in enhancing such parameter in HF, which may carry greater prognostic implications. Interestingly, while this difference remained consistent in HFrEF-only papers, it was not found in the HFpEF analysis, as primarily driven by the recent findings of Mueller et al. [23]. The reason for these contrasting changes is unclear, but may be due to a combination of varying exercise training characteristics and adherence rates, as well as differing underlying physiological mechanisms driving changes in peak VO_2_ in HFrEF and HFpEF. While there are certainly central and peripheral contributions in both phenotypes, in HFpEF patients, chronotropic incompetence and impairments in oxygen extraction and utilisation have been previously considered the primary drivers of exercise intolerance [40], whereas HFrEF is expected to exhibit larger reductions in oxygen delivery due to impaired LV function [41]. As such, the present increase in cardiac systolic function as measured by LVEF, which is supported by previous work [42], may have had a greater influence on peak VO_2_ in HFrEF compared to HFpEF, and therefore, the greater magnitude of increase in LVEF produced by HIIT may have translated into significantly greater improvements in peak VO_2_ for HFrEF, but not HFpEF. Further, these findings should be considered in the context of the magnitude of change, with greater differences seen in HFrEF than HFpEF, potentially contributing to these differing peak VO_2_ results. It is also important to note that HFpEF remains comparatively under-researched in regard to interventional management strategies, and thus the lower number of analysed effect sizes may not have sufficiently powered such statistical analysis, highlighting the need for a greater quantity of rigorous ET trials to truly discern optimal exercise prescription practices in HFpEF. Moreover, given the results of the Egger’s test [21], both the peak VO_2_ and LVEF results should certainly be interpreted with acknowledgement of publication bias. This is especially important given that lower/non-significant effects sizes are reported in the larger scale work analysed within this study [22, 23]. In particular, the extensive studies by Ellingsen et al. [22] and Mueller et al. [23] were the largest trials, also reporting 12-month follow-up data that were excluded from the present analysis but again demonstrated no significant differences in VO_2_ and LVEF changes.

Separately, we found no significant difference across any parameters when MIT is compared against CT. In regard to practical application, this finding provides support for the implementation of CT over MIT alone, indicating that the addition of resistance exercise to MIT does not inhibit the aerobic adaptations in HF. Although these aerobic adaptations are critical to maintain, the addition of resistance training is well established to provide independent metabolic and functional muscular benefits which are essential to offset myopathy and preserve muscular strength [43, 44]. As well as carrying prognostic implications, these muscular adaptations are evidenced to translate into improvements in quality of life [45], which is commonly depleted in both HFrEF and HFpEF [46]. While these findings provide strong support for the inclusion of resistance exercise to ET interventions in HF, very little research to date has investigated the combined effects of HIIT and resistance training, which based on the present analysis, may provide the greatest magnitude of physiological adaptation. A recent pilot study from Hornikx et al. [17] found HIIT supplemented with peripheral resistance and inspiratory muscle training to be more effective than traditional MIT in improving peripheral and inspiratory muscle strength, with no training effect differences in peak VO_2_. Undoubtedly, larger studies are needed to establish the effectiveness of HIIT combined with resistance training in HFrEF and HFpEF cardiac rehabilitation.

### Limitations

Due to various inter-study methodological and interventional differences, significant statistical heterogeneity is a primary limitation of the present analysis. To account for this, random-effects models and meta-regression analyses were applied, but none of the analysed moderators explained any of the observed variance in statistical significance. Additionally, especially in HFpEF, some of the pooled analyses involved a small number of study groups and many of the analysed studies had wide confidence intervals which have implications for the reliability of conclusions drawn from such statistically powered measures. Separately, the majority of analysed studies did not provide sufficient information regarding intravenous iron treatment and its associated benefits, which remains a limitation of current available data. As an inherent limitation to the present work, we collectively analysed patients of differing HF severities, thus conflating the results of patients with heterogeneous baseline characteristics. Furthermore, many HF patients, particularly those randomised to HIIT, often struggle to reach the intensities prescribed in the analysed trials, which may have implications for achieved adaptations. Finally, most included trials do not disclose if the employed protocols are isocaloric or not, and therefore, whether the observed differences disappear when MIT and HIIT protocols are calorie matched is not clear and should be considered.

## Conclusion

HIIT is significantly more effective than MIT for improving peak VO_2_ and LVEF in HF patients. Dichotomized by HF phenotype, HIIT is significantly more effective than MIT for improving peak VO_2_ and LVEF in HFrEF, while only LVEF is significantly improved in HFpEF. Separately, we found no significant differences between MIT and CT for peak VO_2_ or LVEF, suggesting that the addition of resistance exercise to MIT does not impact aerobic adaptations in HF. Based on these findings, future research should investigate the effectiveness of HIIT combined with resistance training to further establish the optimal exercise prescription in HF phenotypes.

## Supplementary Information

**Additional file 1.** Search strategy details, TESTEX study scoring, tabled study characteristic data and advanced moderator analysis results.

## Data Availability

All data generated or analysed during this study are included in this published article and its Additional file 1.
