# Supplementary material for: Exercise Mode in Heart Failure: A Systematic Review and Meta-Analysis
Source: Sports Med Open. 2023 Jan 9;9:3. doi: 10.1186/s40798-022-00549-1 (PMC9829948; doi:10.1186/s40798-022-00549-1)
Supplement: Supplementary file 1 — Additional file 1. Search strategy details, TESTEX study scoring, tabled study characteristic data and advanced moderator analysis results. [file 40798_2022_549_MOESM1_ESM.pdf]

Exercise mode in heart failure: A systematic review and meta-analysis.

Edwards, J.J,[1] Shanmugam, N[2]., Ray, R[2]., Jouhra, F[2]., Mancio, J[2]., Wiles, J.D[1]., Marciniak, A[2]., Sharma, R[2]., O'Driscoll, J.M.[1,2]

[1]School of Psychology and Life Sciences, Canterbury Christ Church University, Kent, CT1 1QU

[2]Department of Cardiology, St George's Healthcare NHS Trust, Blackshaw Road, Tooting, London, SW17 0QT.

Correspondence to Dr Jamie O'Driscoll, School of Psychology and Life Sciences, Canterbury Christ Church University, North Holmes Road, Canterbury, Kent, CT1 1 QU. Email: jamie.odriscoll@canterbury.ac.uk; Telephone: 01227782711.

Supplementary File

Full systematic search

PubMed

Full search: ("exercise"[MeSH Terms] OR “exercise training”[Text Word] OR "cardiac rehabilitation"[Text Word]) AND ("heart failure"[MeSH Terms] OR "heart failure, diastolic"[MeSH Terms] OR HFpEF[Text Word] OR "preserved ejection fraction"[Text Word] OR "normal ejection fraction"[Text Word])

Cochrane

#1 MeSH descriptor: [Exercise] explode all trees

#2 MeSH descriptor: [Cardiac Rehabilitation] explode all trees

#3 #1 OR #2

#4 MeSH descriptor: [Heart Failure] explode all trees

#5 MeSH descriptor: [Heart Failure, Diastolic] explode all trees

#6 HFpEF

#7 "preserved ejection fraction"

#8 "normal ejection fraction"

Web of Science

Set 1: TS=("exercise training") OR TS=("Cardiac rehabilitation")

Set 2: TS=("heart failure") OR TS=("Diastolic heart failure") OR TS=(HFpEF) OR TS=("preserved ejection fraction") OR TS=("normal ejection fraction")

Set 3: WC=Cardiac & Cardiovascular Systems

Search Performed: #1 AND #2 AND #3 (articles only filter applied)

Table S1. Detailed MIT vs CT TESTEX Scoring.

Study name	Eligibility criteria specified	Randomisation specified	Allocation concealment	Groups similar at baseline	Assessors blinded	Outcome measures assessed >85% of participants	Intention to treat analysis	Between group statistical comparisons reported	Point estimates reported	Activity monitoring in control group	Relative exercise intensity review	Exercise volume & energy expended	Overall TESTEX
Laoutaris et al. 2013	YES	NO	NO	YES	YES	YES (2)	NO	YES (2)	YES	YES	YES	YES	11
Beckers et al. 2008	YES	YES	YES	YES	YES	YES (2)	NO	YES (2)	YES	YES	YES	NO	12
Mandic et al. 2009	YES	YES	NO	YES	YES	YES (2)	YES	YES (2)	YES	YES	NO	YES	12
Servantes et al. 2012	YES	YES	YES	YES	NO	YES (3)	NO	YES (2)	YES	YES	YES	YES	13

Table S2. Detailed MIT vs HIIT TESTEX Scoring.

Study name	Eligibility criteria specified	Randomisation specified	Allocation concealment	Groups similar at baseline	Assessors blinded	Outcome measures assessed >85% of participants	Intention to treat analysis	Between group statistical comparisons reported	Point estimates reported	Activity monitoring in control group	Relative exercise intensity review	Exercise volume & energy expended	Overall TESTEX
**Iellamo et al. 2013**	YES	NO	NO	YES	NO	YES (2)	NO	YES (2)	YES	YES	YES	YES	10
**Ellingsen et al. 2017**	YES	YES	NO	YES	YES	YES (3)	YES	YES (2)	YES	YES	YES	YES	14
**Iellamo et al. 2014**	YES	YES	NO	YES	NO	YES (2)	NO	YES (2)	YES	YES	YES	YES	11
**Besnier et al. 2019**	YES	YES	NO	YES	YES	YES (2)	YES	YES (2)	YES	YES	YES	YES	13
**Koufaki et al. 2014**	YES	YES	YES	YES	NO	YES (2)	NO	YES (2)	YES	YES	YES	YES	12
**Wisløff et al. 2007**	YES	YES	NO	YES	NO	YES(3)	YES	YES (2)	YES	YES	YES	YES	13
**Dimopoulos et al. 2006**	YES	YES	NO	YES	NO	YES (1)	NO	YES (2)	YES	YES	YES	YES	10
**Freyssin et al. 2012**	YES	NO	NO	YES	NO	YES (3)	NO	YES (2)	YES	YES	YES	YES	11
**Fu et al. 2013**	YES	NO	NO	YES	NO	YES (2)	NO	YES (2)	YES	YES	YES	YES	10
**Ulbrich et al. 2016**	YES	YES	YES	YES	YES	YES (3)	NO	YES (2)	YES	YES	YES	YES	14
**Donelli da Silveira et al. 2020**	YES	YES	YES	YES	YES	YES (3)	NO	YES (2)	YES	YES	YES	YES	14
**Angadi et al. 2015**	YES	NO	NO	YES	YES	YES (2)	NO	YES (2)	YES	YES	YES	YES	11
**Mueller et al. 2021**	YES	YES	YES	YES	NO	YES (2)	YES	YES (2)	YES	YES	YES	YES	13

Table S3. MIT vs CT participant characteristics.

Paper	Groups	Participants	NYH I	NYH II	NYH III	NYH IV	Ischemic CM	Dilated CM	N. of Males	N. of Females	Mean BMI	Mean age	Beta-blockers %	Diuretics %	ACE inhib %	ARB %	ARB and ACE %	Statin	Digoxin %	Warfarin
**Laoutaris et al. 2013**	MIT	14	0	8	6	0	5	9	12	2	25.9	58.6	71	86	93	NR	NR	NR	64	NR
	Combined MIT + RT + IMT	13	0	6	7	0	4	9	10	3	26.6	57.1	69	92	100	NR	NR	NR	69	NR
**Beckers et al. 2008**	MIT	30	0	12	18	0	18	12	24	6	26.2	59	90	70	NR	NR	100	23	30	40
	Combined MIT and RT	28	0	9	19	0	16	12	18	10	25.7	58	57	85	NR	NR	96	18	39	50
**Mandic et al. 2009**	MIT	14	NR	NR	NR	0	7/14	NR	11/14	3/14	29.8	63	100	71	100	NR	NR	NR	NR	NR
	Combined MIT and RT	15	NR	NR	NR	0	8/15	NR	11/15	4/15	32.1	59	100	93	87	NR	NR	NR	NR	NR
**Servantes et al. 2012**	MIT	18	0	82.4%	17.6%	0	NR	NR	47.1%	52.9%	26.87	51.76	100	100	100	NR	NR	NR	NR	NR
	Combined MIT and SR	18	0	82.4%	17.6%	0	NR	NR	47.1%	52.9%	27.98	50.82	100	82.4	94.1	NR	NR	NR	NR	NR

Table S4. MIT vs HIIT participant characteristics.

Paper	Groups	Participants	NYHA I	NYHA II	NYHA III	Ischemic CM	Dilated CM	Males/Females %	Mean BMI	Mean age	Beta-blockers %	Diuretics %	ACE inhib %	ARB %	ARB and ACE %	Statin	Digoxin %	Warfarin
**Iellamo et al. 2013**	MIT	10	NR	"Mainly NYHA 2"	NR	0	0	100%/0%	27.2	62.6	100%	63%	88%	100%	NR	100%	12.5%	NR
	HIIT	10	NR	"Mainly NYHA 2"	NR	0	0	100%/0%	27.8	62.2	100%	50%	100%	100%	NR	100%	12.5%	NR
**Ellingsen et al. 2017**	MIT	85	0	63%	37%	60%	NR	19%/81%	27.5	60	94%	75%	NR	NR	92%	72%	12%	NR
	HIIT	90	0	71%	29%	60%	NR	18%/82%	27.6	65	95%	75%	NR	NR	92%	65%	22%	NR
**Iellamo et al. 2014**	MIT	18	33.3%	66.7%	0	NR	NR	83.3%/16.7%	28.1	68.4	77.8%	61%	NR	NR	94%	83%	NR	17%
	HIIT	18	44.4%	55.6%	0	NR	NR	88.9%/11.1%	28.3	67.2	88.9%	72%	NR	NR	88.9%	72%	NR	17%
**Besnier et al. 2019**	MIT	15	40%	46.7%	13.3%	11	4	73.3%/26.7%	28	59.5	87%	73%	67%	13%	NR	NR	NR	NR
	HIIT	16	50%	43.75%	6.25%	9	7	68.8%/31.2%	25	59	94%	75%	50%	0%	NR	NR	NR	NR
**Koufaki et al. 2014**	MIT	17	Median 2 (1-3)			NR	NR	76.5%/23.5%	29.2	59.7	59%	65%	76%	NR	NR	53%	NR	NR
	HIIT	16	Median 2 (1-3)			NR	NR	87.5%/12.5%	29.2	59.8	69%	56%	63%	NR	NR	88%	NR	NR
**Wisløff et al. 2007**	MIT	9	NR	NR	NR	NR	NR	77.8%/22.2%	24.7	74.4	100%	44%	100%	NR	NR	100%	NR	67%
	HIIT	9	NR	NR	NR	NR	NR	77.8%/22.2%	24.5	76.5	100%	56%	100%	NR	NR	100%	NR	56%
**Dimopoulos et al. 2006**	MIT	14	28.57%	64.29%	7.1%	5	8	100%/0%	27.2	61.5	86%	79%	21%	NR	NR	NR	NR	NR
	HIIT	10	30%	60%	10%	4	6	90%/10%	26.5	59.2	80%	100%	100%	NR	NR	NR	NR	NR
**Freyssin et al. 2012**	MIT	14	NR	NR	NR	12	14	50%/50%	24.1	55	100%	100%	86%	NR	NR	64%	NR	NR
	HIIT	12	NR	NR	NR	10	12	50%/50%	24.8	54	100%	100%	83%	NR	NR	75%	NR	NR
**Fu et al. 2013**	MIT	15	NR	NR	NR	60%	NR	60%/40%	NR	66.3	93%	47%	NR	NR	80%	NR	27%	NR
	HIIT	15	NR	NR	NR	67%	NR	66.7%/33.3%	NR	67.5	93%	53%	NR	NR	80%	NR	20%	NR
**Ulbrich et al. 2016**	MIT	10	0	100%	0	36.40%	NR	100%/0%	27.47	54.02	45.50%	50%	50%	NR	NR	18.20%	NR	NR
	HIIT	12	0	91.7%	8.3%	31.80%	NR	100%/0%	29.73	53.15	45.50%	40.90%	36.40%	NR	NR	31.80%	NR	NR

**Donelli da Silveira et al. 2020**	MIT	9	0	89%	11%	NR	NR	44%/56%	34	60	78%	89%	NR	NR	100%	78%	NR	NR
	HIIT	10	0	80%	20%	NR	NR	30%/70%	33	60	100%	100%	NR	NR	100%	80%	NR	NR
**Angadi et al. 2015**	MIT	6	0	NR	NR	NR	NR	67%/33%	29.4	71.5	56%	67%	17%	33%	NR	67%	NR	NR
	HIIT	9	0	NR	NR	NR	NR	89%/11%	29.5	69	67%	44%	56%	0%	NR	67%	NR	NR
**Mueller et al. 2021**	MIT	58	0	76%	24%	NR	NR	40%/60%	31.1	70	59%	52%	31%	45%	NR	NR	NR	NR
	HIIT	58	0	76%	24%	NR	NR	29%/71%	30	70	69%	62%	33%	43%	NR	NR	NR	NR

Figure S1: MIT vs CT publication bias funnel plot of standard error by difference in means.

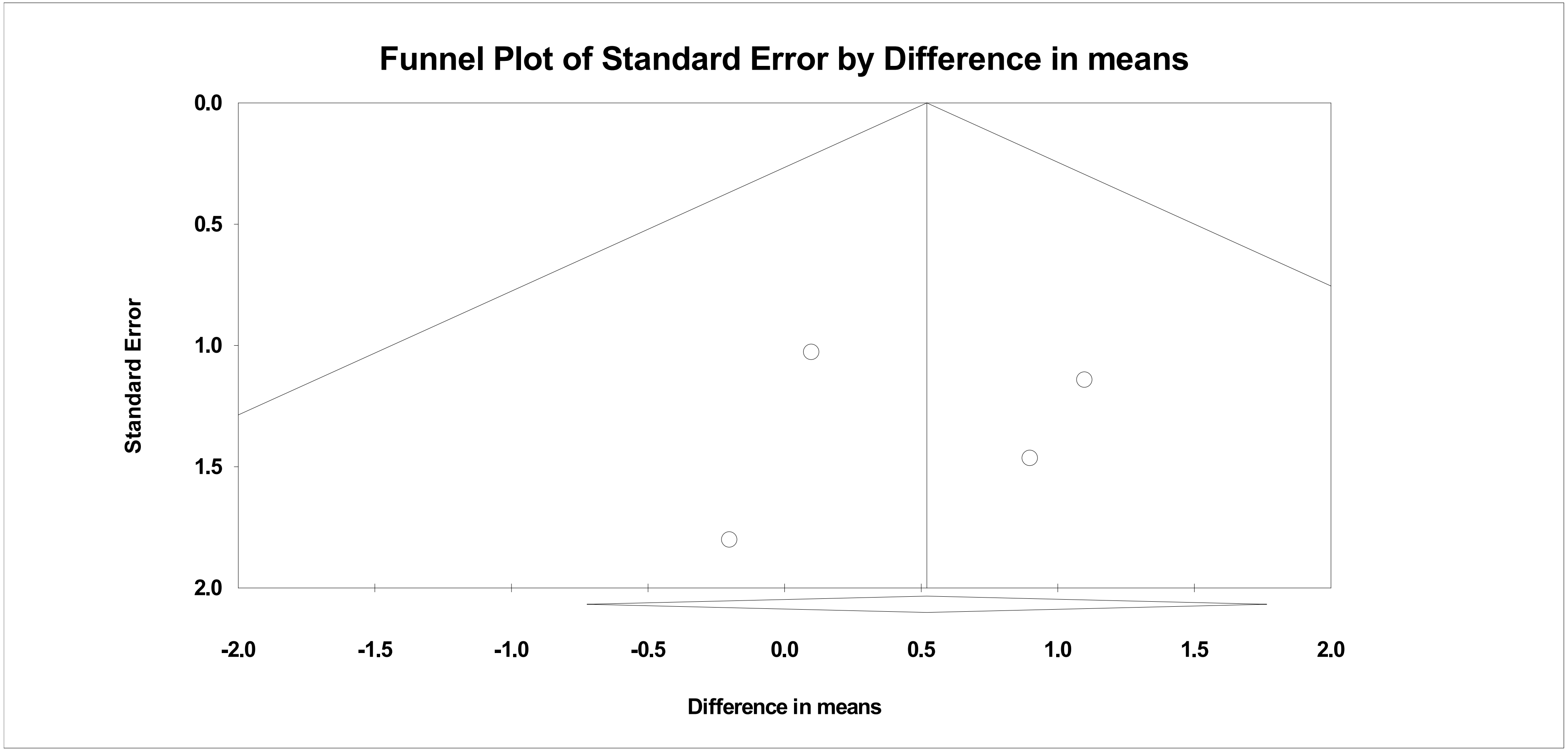

Figure S2: MIT vs HIIT publication bias funnel plot of standard error by difference in me

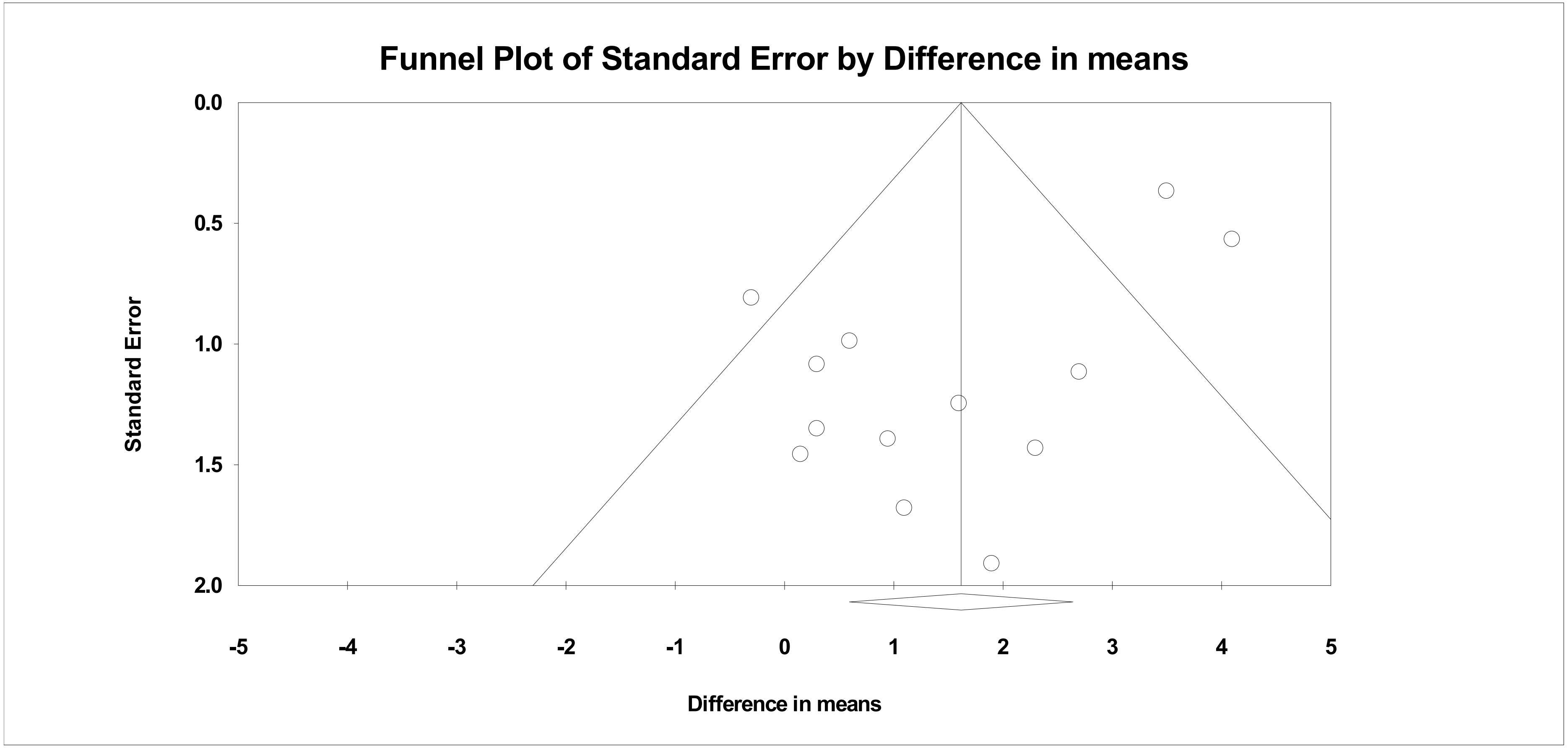

Analysed Studies

MIT vs CT:

1. Beckers, P.J., Denollet, J., Possemiers, N.M., Wuyts, F.L., Vrints, C.J. and Conraads, V.M., 2008. Combined endurance-resistance training vs. endurance training in patients with chronic heart failure: a prospective randomized study. European heart journal, 29(15), pp.1858-1866.
2. Laoutaris, I.D., Adamopoulos, S., Manginas, A., Panagiotakos, D.B., Kallistratos, M.S., Doulaptsis, C., Kouloubinis, A., Voudris, V., Pavlides, G., Cokkinos, D.V. and Dritsas, A., 2013. Benefits of combined aerobic/resistance/inspiratory training in patients with chronic heart failure. A complete exercise model? A prospective randomised study. International journal of cardiology, 167(5), pp.1967-1972.
3. Mandic, S., Tymchak, W., Kim, D., Daub, B., Quinney, H.A., Taylor, D., Al-Kurtass, S. and Haykowsky, M.J., 2009. Effects of aerobic or aerobic and resistance training on cardiorespiratory and skeletal muscle function in heart failure: a randomized controlled pilot trial. Clinical rehabilitation, 23(3), pp.207-216.
4. Servantes, D.M., Pelcerman, A., Salvetti, X.M., Salles, A.F., de Albuquerque, P.F., de Salles, F.C.A., Lopes, C., de Mello, M.T., Almeida, D.R. and Filho, J.A.O., 2012. Effects of home-based exercise training for patients with chronic heart failure and sleep apnoea: a randomized comparison of two different programmes. Clinical rehabilitation, 26(1), pp.45-57.

MIT vs HIIT:

1. Angadi, S.S., Mookadam, F., Lee, C.D., Tucker, W.J., Haykowsky, M.J. and Gaesser, G.A., 2015. High-intensity interval training vs. moderate-intensity continuous exercise training in heart failure with preserved ejection fraction: a pilot study. Journal of Applied Physiology, 119(6), pp.753-758.
2. Besnier, F., Labrunée, M., Richard, L., Faggianelli, F., Kerros, H., Soukarié, L., Bousquet, M., Garcia, J.L., Pathak, A., Gales, C. and Guiraud, T., 2019. Short-term effects of a 3-week interval training program on heart rate variability in chronic heart failure. A randomised controlled trial. Annals of physical and rehabilitation medicine, 62(5), pp.321-328.
3. Dimopoulos, S., Anastasiou-Nana, M., Sakellariou, D., Drakos, S., Kapsimalakou, S., Maroulidis, G., Roditis, P., Papazachou, O., Vogiatzis, I., Roussos, C. and Nanas, S., 2006. Effects of exercise rehabilitation program on heart rate recovery in patients with chronic heart failure. European Journal of Preventive Cardiology, 13(1), pp.67-73.
4. Donelli da Silveira, A., Beust de Lima, J., da Silva Piardi, D., dos Santos Macedo, D., Zanini, M., Nery, R., Laukkanen, J.A. and Stein, R., 2020. High-intensity interval training is effective and superior to moderate continuous training in patients with heart failure with preserved ejection fraction: A randomized clinical trial. European journal of preventive cardiology, 27(16), pp.1733-1743.
5. Ellingsen, Ø., Halle, M., Conraads, V., Støylen, A., Dalen, H., Delagardelle, C., Larsen, A.I., Hole, T., Mezzani, A., Van Craenenbroeck, E.M. and Videm, V., 2017. High-intensity interval training in patients with heart failure with reduced ejection fraction. Circulation, 135(9), pp.839-849.
6. Freyssin, C., Verkindt, C., Prieur, F., Benaich, P., Maunier, S. and Blanc, P., 2012. Cardiac rehabilitation in chronic heart failure: effect of an 8-week, high-intensity interval training versus continuous training. Archives of physical medicine and rehabilitation, 93(8), pp.1359-1364.
7. Fu, T.C., Wang, C.H., Lin, P.S., Hsu, C.C., Cherng, W.J., Huang, S.C., Liu, M.H., Chiang, C.L. and Wang, J.S., 2013. Aerobic interval training improves oxygen uptake efficiency by enhancing cerebral and muscular hemodynamics in patients with heart failure. International journal of cardiology, 167(1), pp.41-50.
8. Iellamo, F., Caminiti, G., Sposato, B., Vitale, C., Massaro, M., Rosano, G. and Volterrani, M., 2014. Effect of High-Intensity interval training versus moderate

continuous training on 24-h blood pressure profile and insulin resistance in patients with chronic heart failure. Internal and emergency medicine, 9(5), pp.547-552.

9. Iellamo, F., Manzi, V., Caminiti, G., Vitale, C., Castagna, C., Massaro, M., Franchini, A., Rosano, G. and Volterrani, M., 2013. Matched dose interval and continuous exercise training induce similar cardiorespiratory and metabolic adaptations in patients with heart failure. International journal of cardiology, 167(6), pp.2561-2565.
10. Koufaki, P., Mercer, T.H., George, K.P. and Nolan, J., 2014. Low-volume high-intensity interval training vs continuous aerobic cycling in patients with chronic heart failure: a pragmatic randomised clinical trial of feasibility and effectiveness. Journal of rehabilitation medicine, 46(4), pp.348-356.
11. Mueller, S., Winzer, E.B., Duvinage, A., Gevaert, A.B., Edelmann, F., Haller, B., Pieske-Kraigher, E., Beckers, P., Bobenko, A., Hommel, J. and Van de Heyning, C.M., 2021. Effect of high-intensity interval training, moderate continuous training, or guideline-based physical activity advice on peak oxygen consumption in patients with heart failure with preserved ejection fraction: a randomized clinical trial. Jama, 325(6), pp.542-551.
12. Ulbrich, A.Z., Angarten, V.G., Netto, A.S., Sties, S.W., Bündchen, D.C., De Mara, L.S., Cornelissen, V.A. and De Carvalho, T., 2016. Comparative effects of high intensity interval training versus moderate intensity continuous training on quality of life in patients with heart failure: study protocol for a randomized controlled trial. Clinical Trials and Regulatory Science in Cardiology, 13, pp.21-28.
13. Wisløff, U., Støylen, A., Loennechen, J.P., Bruvold, M., Rognmo, Ø., Haram, P.M., Tjønna, A.E., Helgerud, J., Slørdahl, S.A., Lee, S.J. and Videm, V., 2007. Superior cardiovascular effect of aerobic interval training versus moderate continuous training in heart failure patients: a randomized study. Circulation, 115(24), pp.3086-3094.
